# Surface alignment of nematic liquid crystals by direct laser writing of photopolymer alignment layers

**DOI:** 10.1080/02678292.2023.2242297

**Published:** 2023-08-07

**Authors:** Uroš Jagodič, Mahendran Vellaichamy, Miha Škarabot, Igor Muševič

**Affiliations:** a Condensed Matter Department, J. Stefan Institute, Ljubljana, Slovenia; b Faculty of Mathematics and Physics, University of Ljubljana, Ljubljana, Slovenia

**Keywords:** 3D printing, direct laser writing, alignment layer, surface patterning

## Abstract

We demonstrate the fabrication of good quality surface alignment layers on glass by Direct Laser Writing method using a 2-photon polymerisation technique. We use commercially available photosensitive resins to print alignment layers by scanning the focal point of a femtosecond laser near the resin-glass interface. This results in down to ~ 100 nm thin alignment layers that provide good planar anchoring of 5CB and MLC13300, with the easy axis of alignment along the scanning direction. The azimuthal anchoring strength is ~ 5 × 10^−6^ J/m^2^ and is an order of magnitude weaker compared to commercial rubbed polyimide alignment layer. The threshold voltage for Fréedericksz transition in a 90° twisted nematic cell is slightly increased compared to conventional rubbed polyimide for printed alignment layers. The turn-on switching time is longer for printed layers compared to polyimide alignment layers, whereas the turn-off time is shorter for printed alignment layers. The advantage of this new method is in its flexibility, as we demonstrate printing of complex surface alignment patterns with alignment layer thickness below 100 nm.

## Graphical abstract

**Figure:**
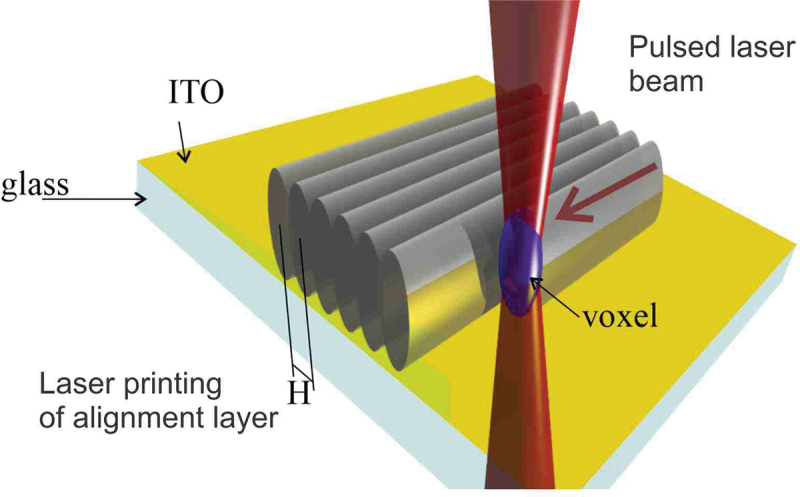

## 1. Introduction

The control of surface anchoring of nematic liquid crystals (NLCs) is not only of great technological importance but is also crucial for many fundamental studies of LC surfaces and interfaces [1]. Since the pioneering work of Lehman [2], Grandjean [3], Mauguin [4], and Chatelain [5], a great number of studies have been performed on surface alignment of liquid crystals, and there is good understanding as of why and how the NLCs align on a particular surface [6].

There are several physical mechanisms that are responsible for the surface anchoring of NLCs. It is known since the experiments of Berreman [7,8] that nano-sized grooves fabricated on the surface of a solid substrate align the NLC director in the bulk parallel to that grooves. Such grooves can be produced either by mechanical rubbing of a solid surface or by patterning thin organic films on substrates by the stylus of an Atomic Force Microscope (AFM) [9–14]. Bistable and multistable surface orientations of a NLC could be obtained on specially designed surface patterns engraved by the AFM [15,16], mimicking the multiply stable surface orientations of a NLC on freshly cleaved surfaces of some single crystals like mica [11,17,18]. Surface pretilt angle of a NLC on AFM-micro-textured surfaces can be controlled by varying the grooves’ spacing [19]. Another well-known mechanism of controlled surface alignment is based on direct intermolecular interactions and molecular epitaxy of the LC molecules on the surface. For example, cyano-biphenyl NLCs show good planar alignment on graphite, and it was demonstrated by Scanning Tunnelling Microscopy [20] that the ordering of 8CB molecules on graphite is crystalline-like in the first molecular layer. The mechanism of alignment of NLCs on mechanically rubbed or photo-polymerised interfaces is similar to the alignment on solid crystalline surfaces. It has been shown that mechanical rubbing produces of a polymer surfaces create a very thin layer of the polymer that has been aligned on the molecular scale by the rubbing process [21,22]. This aligned surface layer then acts as an aligning matrix for NLC molecules in bulk. A very powerful method of LC alignment is photo-alignment [23,24], where a UV linearly polarised light and selective chemical reactions are used to create a degree of ordering in the surface of a photosensitive alignment layer. Recently, Guo et al. [25] developed a novel technique for photo patterning of surfaces using plasmonic meta-masks that generates spatially varying polarisation direction of light with high-resolution.

Quite recently, the alignment of NLCs on surfaces, printed by two-photon polymerisation Direct Laser Writing (DLW), has attracted a lot of attention. Zhichao et al. [26] used a focused beam of a femtosecond laser beam at λ = 800 nm to fabricate thin polymer ribbon patterns of SU-8 3025 photoresist on glass in a single scan. They observed that a pair of thin (several μm) and ~10 μm tall polymer ribbons, separated by some tens of micro-metres, could align very well the nematic liquid crystal in between. It was found that the NLC alignment is due to the fine relief of surface of the vertical walls of the ribbons. The periodicity of these surface undulations was ~250 nm, which equals to one-half of the wavelength of the laser light in the SU-8 film. This suggests that the formation of the periodic surface relief structure on the sidewalls of the ribbons came from the optical interference between the incident beam and the reflected beam by the ITO film between the glass substrate and the SU-8 film. In another study, Yuping Shi et al. [27] used polarised direct laser writing to encode preferential alignment of polarisation sensitive photo-alignment layers to align the NLC in contact with this layer. Further on, O’Neill et al. [28] fabricated 3D diffractive and switchable optical micro-components by two-photon polymerisation of a polymer NLC. They took advantage of the orientational ordering of bulk polymer nematic crystal to encode not only 2D but also 3D alignment of NLC on polymer microstructures. This made it possible to produce 3D multi-element computer generated holograms that could be switched by external voltage.

In this article, we demonstrate that a single DLW printed polymer layer on glass substrate provides an excellent planar alignment of pentyl-cyano-biphenyl (5CB) and MLC13300 (Merck). We assembled 90 degrees twisted nematic cells with printed alignment layer on one of the glass slides, whereas the other slide was covered with a rubbed polyimide alignment layer. Good optical contrast of ~ 1:30 was obtained for the TN cell between parallel and crossed polarisers, respectively. We measured the azimuthal surface anchoring strength on a single DLW printed layer, which is ~ 5 × 10^−6^ J/m^2^. We performed measurements of the threshold voltage for Fréedericksz transition in 90° TN cells, we determined the switching times for the electrooptic response and compared the electro-optic (EO) performance of TN cells made with rubbed polyimide alignment layer and DLW printed layers. Finally, we demonstrated printing of complex surface alignment layers with sub-micron details, which shows the full potential application of this new alignment method.

## 2. Experimental methods and materials

**Figure 1.**
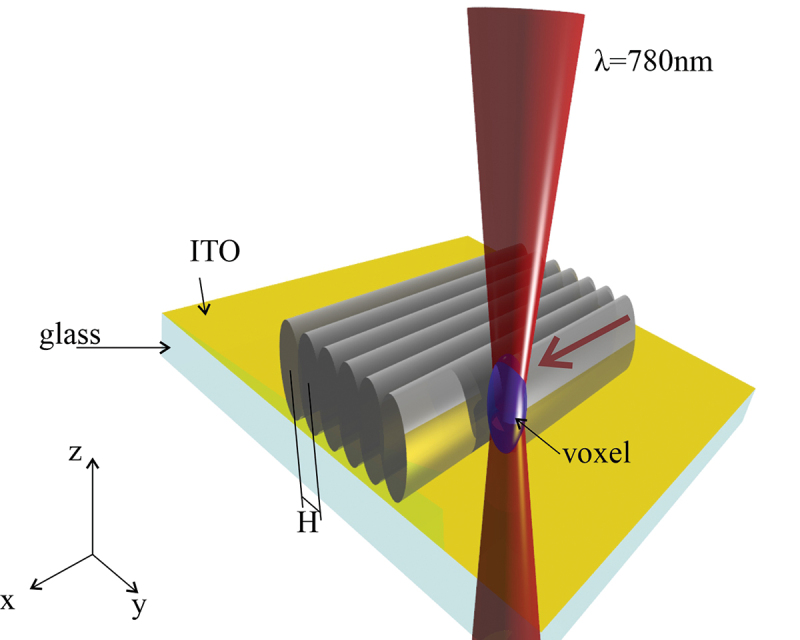
(Colour online) Schematic representation of the two-photon direct laser writing (DLW). The blue spheroid marks the voxel area in which the light intensity is above the threshold for two-photon polymerisation and thus polymerisation can occur. The distance between two lines printed by the movement of the voxel in the x-y plane is called hatching (***H***). The red arrow indicates the direction of movement of the laser focus.

Surface alignment layers are fabricated using a direct laser writing system (Photonic Professional GT2, Nanoscribe GmbH). A high-powered femtosecond laser beam of 780 nm is moved at the interface between a photosensitive resin and ITO layer on glass slide, as shown in Figure 1. The power of the laser is adjusted to the threshold level, where the two-photon polymerisation of the resin occurs only in the focal plane of the beam, where the peak laser power is high enough to reach the two-photon polymerisation threshold. The cross-section of the polymerised volume in the focal plane – a voxel – depends on the objective used. For 63×, 1.43 NA objective, the size of voxel cross-section in the x-y plane is ~120 nm and ~300 nm in the z direction. For for 25×, 0.9 NA objective, the voxel size is 600 nm in the x-y plane and 3 μm in z-direction.

We used 150 μm thick glass slides with 30 nm ITO coating (produced by Diamond Coatings Ltd), which provides good optical contrast of the glass-IPS resin interface for an efficient auto-focusing of the Nanoscribe printer. The substrates are thoroughly cleaned with lint free cloths, isopropanol and acetone, after which they are sonicated for 30 min in detergent (Hellmanex III, Hellma Analytics) baths. After sonication, the substrates are thoroughly rinsed with DI water and dried in an isopropanol vapour degreaser. To enhance the adhesion, the glasses are then baked at 110°C for 30 min to remove surface water and are further placed under a UV plasma for 5 min to remove any organic material. Immediately after the plasma treatment, a small amount of IP-S/IP-L/IP-n162 resin (Nanoscribe GmbH) is drop-casted onto the glass substrate. The so-prepared substrate is glued onto the substrate holder and placed into the GT2 Photonic Professional for printing.

We use a dip-in printing mode, where the space between the bottom surface of a glass slide and the lens of a microscope objective of the printer is filled with a photosensitive resin. The printing starts after the autofocus determines the ITO glass-resin interface. During the printing, the voxel is moved in the focal plane of the objective using the galvo mirrors, until the first layer is printed line by line. The use of galvo mirrors allows for printing speeds of up to several 100 mm/s, depending on printing parameters. The spacing between the neighbouring lines is called the hatching, indicated by ‘H’ in Figure 1(a). We tested several different photosensitive resins: (i) a high resolution, low shrinkage IP-L resist, (ii) a smooth structural surface finish IP-S resist, and (iii) a high refractive index IP-n162 resist, all of them are produced by Nanoscribe GmbH.

**Figure 2.**
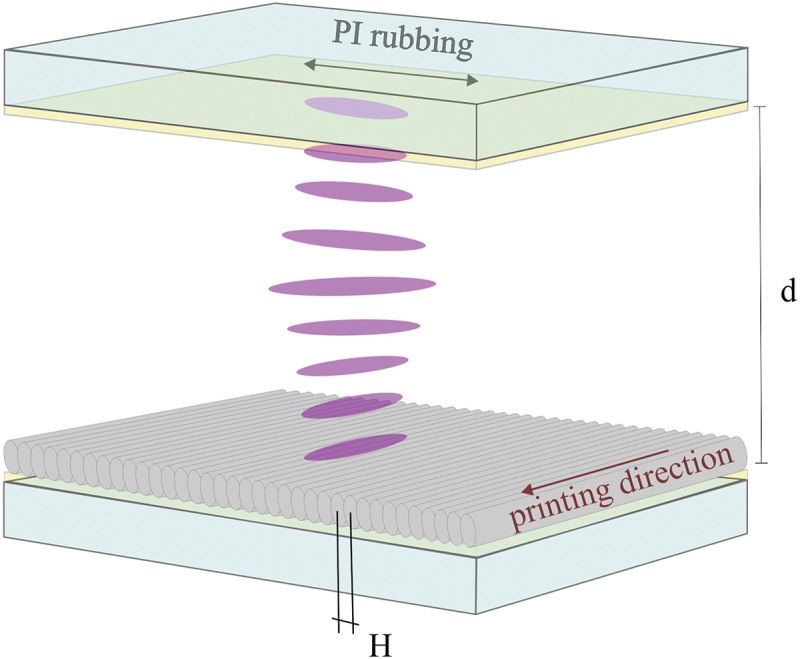
(Colour online) Structure of a 90° twisted nematic cell used in optical and electrooptical experiments. The direction of DLW printed grooves on lower glass and the rubbing direction on polyimide on upper glass are orthogonal. H denotes the hatching distance and d is the thickness of the cell.

After the printing, the substrates are submersed into SU8 developer for 20 min and then washed for 5 min in isopropanol, which completely dissolves al the unpolymerised photoresist and leaves the printed structures on the substrates. We analysed the quality of NLC alignment for each of the resins used. The surface topography of the 2D printed single polymer layers was imaged using an Atomic Force Microscope (AFM, Nanoscope IIIa, Digital Instruments, USA) in the tapping imaging mode. Two different types of LC cells were assembled for the experiments: (i) for surface anchoring measurements, the cells were assembled from one glass slide that was DLW patterned and another glass slide that was covered with a 20 nm layer spin-coated Polyimide (PI 5291 Nissan, Japan) and unidirectionaly rubbed with a fine cloth. The structure of the cell is shown in Figure 2, presenting the direction of DLW printed grooves on one glass plates and the PI rubbing direction on the other glass plate. The cell thickness was determined by 8 μm glass spacers, and the cell was filled with 5CB LC by capillary action. (ii) The cells for electrooptic threshold and switching time measurements were assembled using two identical glass slides that were both either DLW-patterned or covered with polyimide and rubbed. The thickness of these cells was controlled by 18 μm glass spacers, and the cells were filled with MLC13300 nematic LC by capillary action. All the cells were inspected under an upright polarising microscope (Nikon Ecllipse e600 POL), equipped with Canon EOS 550D camera. The static optical contrast of the cells was determined by directly reading the voltage level of individual pixels of the camera. The threshold voltage was measured by reading the voltage of individual pixels on the camera at different applied AC voltage of 1k Hz frequency. The electrooptic response of the cells was measured under the optical microscope by reading the voltage signal from a photodiode, capturing the time-dependence of the transmitted light intensity, and stored in a digital oscilloscope (Infinium MSO9404A, Agilent Technologies, Inc.).

The azimuthal (in-plane) anchoring strength of 5CB on single layer printed alignment IPS layers was measured by using the method described in Ref [29]. Briefly, 90° twisted nematic regions were produced in a wedge-type cell by printing several well-separated boxes. For a selected position in the wedge cell, the local thickness of the cell is *d*, and the actual twisting angle Φ_TN_ of the nematic at that thickness is less than 90°. The smaller the thickness *d* of the NLC layer, the smaller is the total twisting angle Φ_TN_, because the torque due to twisting of the nematic increases with decreasing thickness of the twisted nematic. For a given thickness *d*, the angle Φ_TN_(d) is determined optically and the azimuthal anchoring strength is calculated [29].

## 3. Results

**Figure 3.**
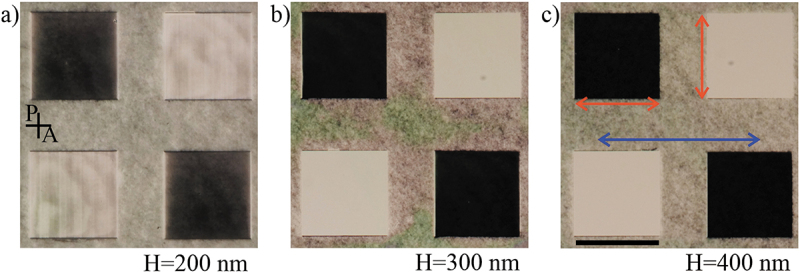
(Colour online) (a–c) Polarizing microscope image of an 8 μm thick cell filled with 5CB doped with 0.1% of CB15 and between crossed polarisers, taken for different hatching distances of the DLW printing process using the IPS resin and 25 × objective. The printed glass plate was cleaned after printing and was not treated for any special surface alignment, thus showing random planar alignment in areas that were not printed. The second glass plate of the cell was covered with a 20 nm layer of PI and rubbed in one direction. The red line shows direction of laser printing and the blue line shows direction of PI rubbing, scale bar is 50 μm.

We printed chequerboard patterns of a single layer of IPS on ITO glass slides, using 25× objective, as shown in Figure 3(a–c) The direction of the laser writing (e.g. moving the focus) on individual fields of the chequerboard is shown in Figure 3(c) and alternates from down-up to right-left in neighbouring squares. The chequerboard patterns were printed using different hatching distances from H = 100 nm to H = 500 nm, and we found that good quality alignment of the NLC was obtained for a limited range of H between 200 and 500 nm only. The LC used to fill the cells is 5CB doped with 0.1% wt% of chiral dopant CB15 to remove the twist degeneracy of 90° TN cells and appearance of reverse twist domains.

**Figure 4.**
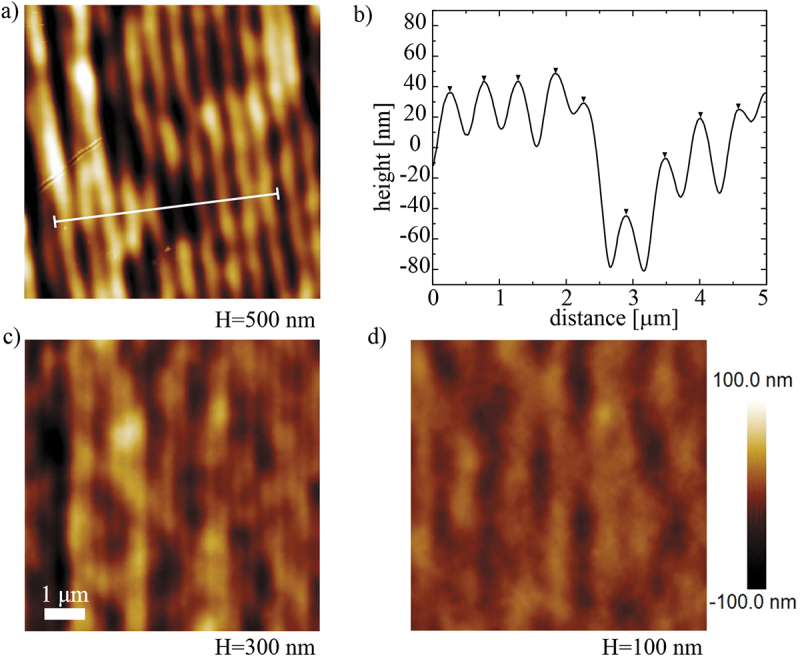
(Colour online) (a,c,d) AFM images taken on selected surfaces of chequerboard, printed with IPS resin at various hatching distances H. (b) Cross section of surface topography along the white line in Figure 3(a). Vertical scale bar that indicates the height, is the same for all images. Scale bar 1 μm.

It is clearly seen from Figures. 3(a–c) that the DLW printed surface indeed promotes good planar alignment of a nematic LC. The direction of alignment of the NLC on printed surface coincides with the direction of scanning of the laser focus. The surface topography and the thickness of a single DLW printed layer of IPS resin, printed with 25× objective, was analysed by taking AFM images at different places on the DLW printed areas and are shown in Figure 4 for hatching distances H = 500, 300, and 100 nm, respectively. The printing direction is approx. vertical in all panels of Figure 4. When the hatching distance H is 500 nm, one can clearly resolve vertical grooves, as shown in Figure 4(a). The height of the grooves and the distance between them can be resolved by taking a cross-section in Figure 4(b), which is along the direction, indicated by the white line in Figure 4(a). The measured distance between the neighbouring maxima in Figure 4(b) is ∼ 500 nm and matches the hatching distance of H = 500 nm used for printing. Similar, but shallower, grooves with the distance 300 nm can be observed in the sample with the hatching distance of H = 300 nm (Figure 4(c)). However, when the hatching distance is H = 100 nm (Figure 4(d)), grooves with hatching separation cannot be resolved any more. Here, one can resolve shallow grooves with approx. 1 µm distance. We assume that these shallow grooves appear due to the refraction and focusing of the laser light on the already polymerised resin, which has higher refraction index than the unpolymerised resin and acts as a waveguide. According to all these observations, we conclude that the good alignment is achieved by deep surface grooves produced by printing. The additional shallow grooves with approx. 1 µm lateral periodicity could be responsible for rather weak alignment at H = 100 nm.

We should note that by using 63× objective for printing, surfaces with less pronounced grooves with hatching periodicity were observed. However, these alignment layers also resulted in good quality twisted nematic cells. This indicates two different mechanisms of azimuthal surface anchoring on printed polymer surfaces: (i) parallel grooves are visible in AFM images at higher hatching distances and are responsible for azimuthal anchoring along the Berreman’s model. (ii) In addition, there must be another mechanism that is effective on a nanoscale, indicating local polymer chain alignment due to the voxel movement along the printing direction.

The thickness of the printed alignment layers depends on the objective used for printing because different objectives have different voxel sizes. When 25× objective was used, the minimum thickness of printed layers was ~1100 nm. When the layers were printed with 63× objective, the thickness of the alignment layer was reduced to ~100 nm. This is due to ~300 nm long voxel in z-direction (along the beam direction) when using 63×, 1.43 NA objective, compared to 3000 nm long voxel for 25×, 0.9 NA objective.

**Figure 5.**
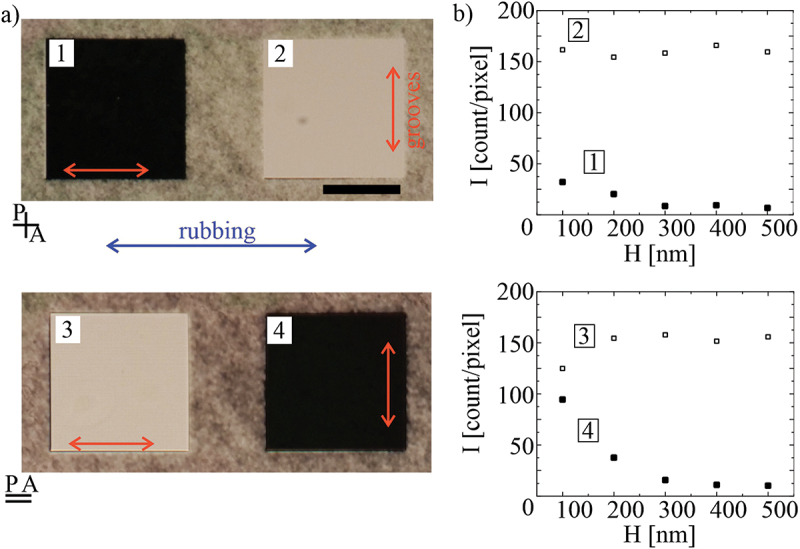
(Colour online) Optical contrast of the IPS chequerboard printed at different hatching distances H = 100 to 500 nm. (a) Photo of a pair of squares with hatching distance H = 400 nm of the chequerboard alignment layer in an 8 μm thick cell, filled with 5CB between crossed polarisers in squares 1 and 2. The same squares between parallel polarisers are shown in 3 and 4. The IPS resin was used for printing the alignment layer, the red arrows indicate the printing direction on each square. The blue arrow indicates the rubbing direction of the PI layer on the second glass of the cell. Scale bar 50 μm. (b) the intensity of white light, transmitted through the squares for different settings 1–4, measured for different hatching distances 100–500 nm.

The quality of surface alignment of a NLC on DLW IPS printed surfaces was characterised by measuring the contrast ratio of a chequerboard pattern in Figure 5(a). Using a video camera, we measured the intensity of light transmitted through each of the squares of the chequerboard pattern for parallel and crossed polarisers, respectively. The corresponding light-levels, as measured by the camera, are shown in Figure 5(b) for different hatching distances. It is clearly seen that below H = 300 nm, the contrast between the dark and bright field becomes small, whereas for H >300 nm, the contrast ratio I^bright^/I_dark_ is larger than 30 for the IPS printed patterns.

**Figure 6.**
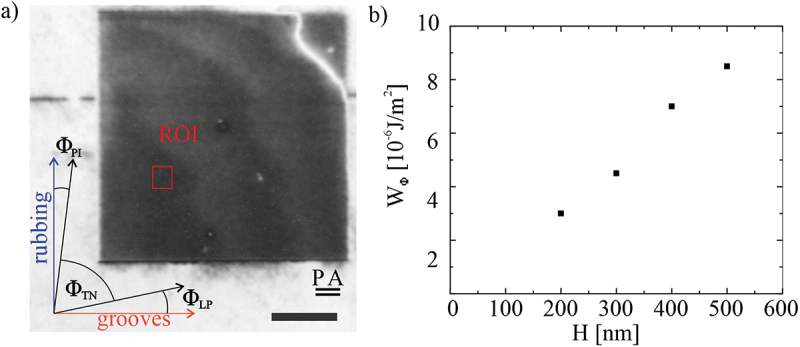
(Colour online) The strength of azimuthal orientational anchoring of 5CB on printed IPS alignment layer. (a) a 5CB cell made of one glass with rubbed PI and another glass with printed IPS grooves, between parallel polarisers. ROI indicated the region, where the measurements of azimuthal anchoring were performed. The angles Φ_PI_, Φ_TN_ and Φ_LP_ are defined in lower left corner of this panel. Scale bar 25 μm. (b) the azimuthal anchoring strength for IPS printed alignment layer as a function of hatching distance H.

We also measured the in-plane (azimuthal) anchoring strength of 5CB on a single layer printed alignment layers of IPS resin. We used an optical method, described in Ref [29]. The method was first tested on a wedge-type, 90° twisted nematic cells with PI alignment layers and filled with 5CB. A NLC cell was made in a form of a wedge, with the thickness varying from 2 to 14 μm. The cell was filled with 5CB and placed under an upright Nikon microscope equipped with white LED illumination (CoolLED pe-100) and a 10 nm bandpass filter centred at 550 nm to obtain reasonably narrow spectrum of measuring light. The intensity of light, passing through a rotatable polariser, the wedge-like TN cell and another rotatable analyser, was detected by a camera (PixeLINK PL-A741). The sample was placed at the focal plane of the microscope, thus obtaining a sharp image of the TN structure between two polarisers. The measurement of the in-plane anchoring strength involves several synchronised rotations of the polariser, the cell, and the analyser, to obtain the position of the polariser and the analyser, where the transmitted light level reaches an absolute minimum [29]. In this position, the polariser is at an angle Φ_PI_ with respect to the rubbing direction and the analyser is at the same angle -Φ_PI_ with respect to the same direction, as presented in Figure 6(a). The actual twisting angle Φ_TN_ of the nematic structure is less than 90° because the director at each interface deviates from the rubbing direction for an angle Φ_PI_ due to elastic torque of the twisted nematic LC that wants to keep the NLC untwisted. The angle Φ_PI_ is very small for large thickness of the cell (lower elastic deformation) and increases by decreasing the thickness of the cell. For two equal PI rubbed surfaces in a 90° TN cell of 8 μm thick 5CB, the deviating angle Φ_PI_ equals ~ 2°. From this value of the deviating angle α and taking the twist elastic constant of 4.1 pN for 5CB, we calculated the azimuthal anchoring strength of 4 × 10^−5^ J/m^2^ for 5CB on rubbed PI. Now, after we have calibrated our method of measuring the azimuthal anchoring strength on PI rubbed surface, we proceeded with measurements of azimuthal anchoring strength on DLW printed alignment layers with various hatching distances. Using the same method, we have measured the deviation angle Φ_LP_ between the direction of laser printed grooves and the director at the laser printed layer on first substrate and deviation angle Φ_PI_ on the other substrate, see Figure 6(a). Using the deviation angle Φ_LP,_ we can determine the azimuthal anchoring strength for IPS printed resin for various hatching distances, which is in the range from 3–8 × 10^−6^ J/m^2^ and is shown in Figure 6(b). It is therefore almost an order of magnitude weaker azimuthal anchoring for 5CB on DLW printed layers than on rubbed PI. We should note that the azimuthal anchoring strength on DLW printed alignment layers is comparable to the measurements of Vilfan et. al [30]. They use Dynamic Light Scattering in different scattering geometries on wedge-type nematic cells to determine both zenithal and azimuthal surface anchoring coefficients of 5CB on rubbed poly-(vinyl-cinnamate) (PVCi) and nylon. They measured azimuthal anchoring coefficient of 1.4 × 10^−5^ J/m^2^ for 5CB on rubbed nylon and 5.6 × 10^−5^ J/m^2^ for 5CB on rubbed PVCi. This is comparable to the azimuthal anchoring coefficient that we measured for PI, and almost an order of magnitude stronger compared to azimuthal anchoring on DLW printed IPS alignment layers.

The azimuthal anchoring coefficient for 5CB on grooved surfaces can be also estimated and compared to the Berreman’s model [7,8] for azimuthal surface anchoring strength W_Φ_ on grooved surfaces:

(1) $$W_{\Phi }=\frac{4\pi^{3}Kh^{2}}{p^{3}}$$

Here, *h* is the amplitude and *p* is the periodicity of grooves, whereas *K* is the arithmetic mean of the Frank constants of nematic LC. For a hatching distance H = 500 nm, the amplitude is *h* = 25 nm, the periodicity is *p* = 510 nm (as measured by the AFM) and by taking the average Frank elastic constant K = 6.6 pN for 5CB, we obtain surface anchoring coefficient *W*_*Φ*_ = 4 × 10^−6^ J/m^2^. This is somewhat, but not significantly, lower than optically determined value on 90° TN cells. We assume that this difference is due to the other nanoscale alignment mechanisms, which were previously discussed.

**Figure 7.**
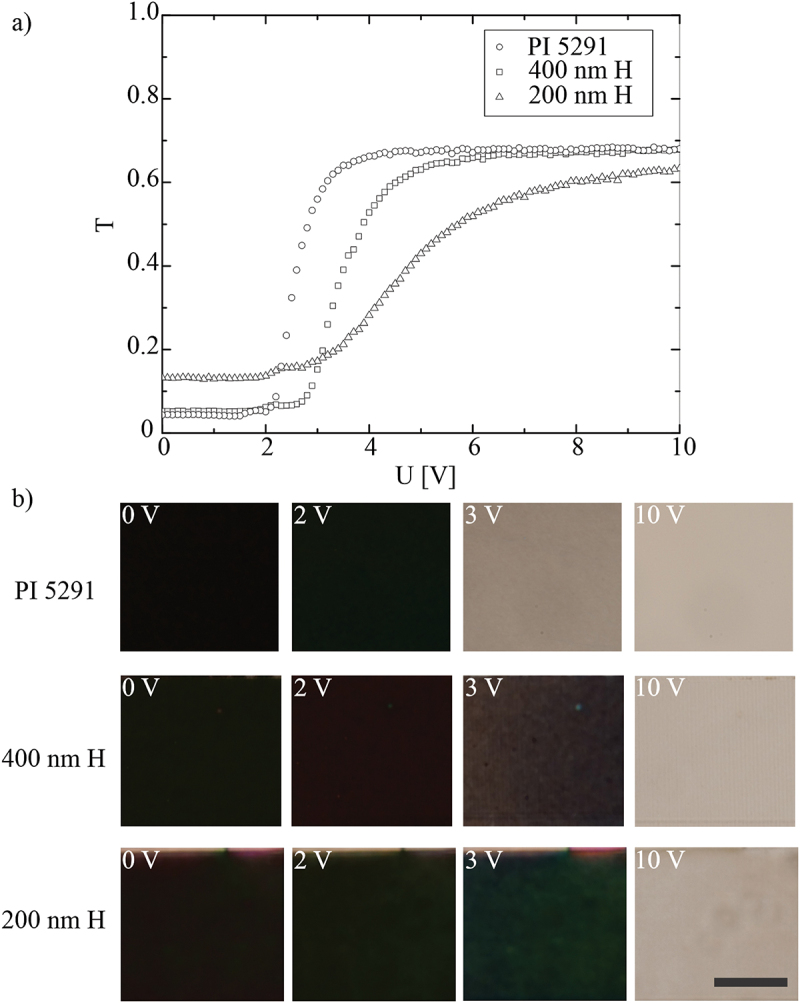
(Colour online) Static electrooptic properties of 90° TN cells with DLW printed alignment layers. (a) Measurements of the threshold for Fredericksz transition in a 18 μm 90° TN cell, filled with MLC13300 nematic mixture (Merck) with small amount of chiral dopant MLC6247 (Merck) added. Three different cells were assembled with different alignment layers, as described in the main text. (b) the texture of the different TN LC cells between crossed polarisers in the vicinity of the threshold voltage (2 V to 3 V AC) and in the saturated regime at 10 V AC. Scale bar 50 μm.

After determining the azimuthal anchoring strength of DLW printed alignment layers, we measured the electrooptic characteristics of 90° TN cells, assembled with two identical DLW alignment layers (on upper and lower glass of Figure 2) and with orthogonal directions of grooves on each glass plate. For comparison, we made identical experiments with the cells that had rubbed polyimide PI 5291 alignment layer. Figure 7(a) shows the measurements of a threshold voltage for 90° TN cell for 3 different cells: (i) 18 μm thick TN cells with rubbed PI layer on both glass plate (empty circles), (ii) 18 μm thick TN cells with 400 nm hatching distance DLW printed grooved layers on both glass plates (open squares) and (iii) 18 μm thick TN cells with 200 nm hatching distance DLW printed grooved layers on both glass plates (open triangles). One can clearly see that the threshold voltage and the contrast ratio of the cells made of 400 nm hatching distance DLW printed alignment layers are very similar to the rubbed PI 5291 cells, whereas the 200 nm hatching distance gives higher threshold voltage and lower optical contrast between the dark (off) state and the bright (on) state. For 400 nm hatching distance DLW printed layers, there is a slight (~20%) increase of the threshold voltage compared to cell with PI 5291 alignment layers, but with practically the same slope. One can clearly see a slight difference in the contrast ratio for PI-PI and DLW-DLW cells. The contrast ratio the cell with two PI alignment layers is slightly higher compared to the cell with two DLW400 printed layers, see Figure 7(a). Microscope images of the three types of cells are shown in Figure 7(b). In all cases, there is good optical quality of the NLC structure throughout the region of switching between the twisted nematic below the threshold and vertically aligned NLC texture above the threshold for Fréedericksz transition.

**Figure 8.**
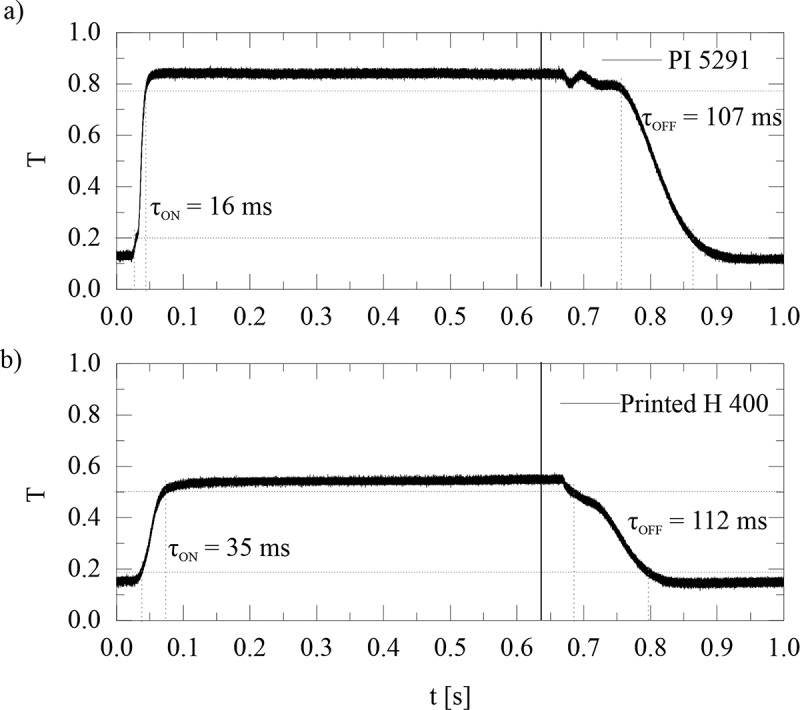
(Colour online) Response times of cells with DLW printed alignment layers compared to TN cells with PI alignment layers. (a) the electro-optic response of a 18 μm cell with two rubbed PI alignment layers, filled with MLC13300 NLC to 10 V amplitude electric pulse of 630 ms duration. The frequency of the applied voltage is 1 kHz. (a) the electro-optic response to 10 V_pp_ electric pulse of 630 ms duration of a 18 μm cell with two DLW H = 400 alignment layers, filled with MLC13300 NLC. The frequency of the applied voltage is 1 kHz.

We also characterised the switching times of the TN cells prepared with PI 5291 in DLW400 alignment layers. Figure 8 shows the measurements of response times τ_on_ and τ_off_ for DLW400 18 μm TN cell (Figure 8(b)) and PI 5291 18 μm TN cell (Figure 8(a)). In both cases, a square wave voltage of 10 V amplitude and 1 kHz frequency was applied at *t* = 0, and the duration of the pulse burst was 630 ms. The turn-on time for DLW alignment layers was doubled (~35 ms) compared to PI alignment layers (~16 ms), whereas the turn-off time for DLW layers was comparable (~112 ms) to the PI layers (~107 ms). However, one can clearly see that the time delay between the end of the electrical pulse and the start of decay is much shorter for DLW printed layers.

These results demonstrate that DLW printed alignment layers are somewhat, but not significantly, inferior to polyimide alignment layers in terms of dynamic response. Most probably this is due to additional elastic energy of the NLC that is stored in the elastically distorted NLC inside the grooves. Whereas, this stored energy prevents fast turn-on, it facilitates turn-off of the TN structure.

**Figure 9.**
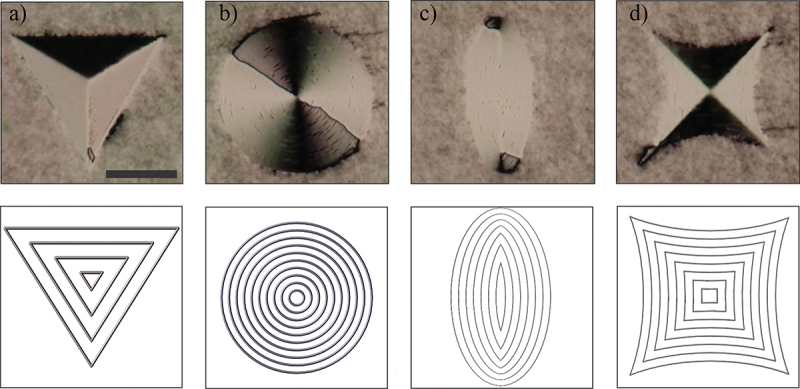
(Colour online) (a-d) Complex alignment patterns, printed with IPL resin on glass with hatching distance H = 400 nm. The thickness of 5CB cell was 8 μm, the other glass was rubbed PI, photos were taken between crossed polarisers. Scale bar 50 μm.

Most importantly, the DLW printed alignment layers clearly provide an advantage in terms of flexibility of surface patterning. For example, Figure 9 shows several complex patterns that illustrate the capability of new method of printing NLC alignment layers. In triangular pattern in Figure 9(a), the direction of laser writing is along the sides of the triangle. In circular pattern in Figure 9(b), the laser focus was moved in concentric circles, whereas in elliptic and hyperbolic patterns in Figs. 9(c,d), the laser focus was moved in concentric ellipses and hyperbolas starting from the outer edge towards the centres. The printing patterns are shown in panels below each photo.

## 4. Conclusions

We demonstrate that alignment layers can be rapidly printed on glass using 2-photon, direct laser writing technique. The alignment layers are of good optical quality and exhibit a moderate azimuthal anchoring strength of ~ 3–8 × 10 ^−6^ J/m^2^ for NLCs. The threshold voltages for Fréedericksz transition are slightly increased for DLW printed layers compared to conventional rubbed polyimide. While the turn-on time is nearly doubled for the particular NLC (MLC13300) used in DLW cells, the turn-off time is reduced due to shorter delay time for the beginning of elastic relaxation. The most important advantage of direct writing of LC alignment layers is the flexibility of alignment pattern design and could be used to produce complex patterns for application in micro-photonics with small footprint. Furthermore, the printing speed is significant, and 100 × 100 μm^2^ patterned alignment layers could be printed in less than 10 seconds. This new method is not only useful to produce planar, e.g. 2D patterns, but could be used to print alignment patterns in 3D. This will be demonstrated in the near future.
